# Single‐stage ACL reconstruction and displaced bucket handle Meniscus repair is associated with lower Meniscus repair failure rates compared to two‐stage surgery

**DOI:** 10.1002/jeo2.70199

**Published:** 2025-03-07

**Authors:** Carolina Kekki, Riccardo Cristiani, Anders Stålman, Christoffer von Essen

**Affiliations:** ^1^ Department of Molecular Medicine and Surgery Section of Sports Medicine, Karolinska Institutet Stockholm Sweden; ^2^ Stockholm Sports Trauma Research Center (SSTRC), FIFA Medical Centre of Excellence Stockholm Sweden

**Keywords:** ACL reconstruction, bucket‐handle, meniscus injury, meniscus repair

## Abstract

**Purpose:**

To compare displaced bucket‐handle meniscus repair (BHMR) failure rates, subjective and objective knee function after BHMR in the setting of ACLR performed as a single‐or two‐stage procedure, and assess factors associated with BHMR survival.

**Methods:**

This retrospective study included patients who underwent surgery between February 2015 and December 2021 at one institution. Patients with a displaced bucket‐handle meniscus tear (BHMT) and ACL‐injury undergoing BHMR and ACLR as a single‐ (concomitant BHMR and ACLR) or two‐stage (BHMR and subsequent ACLR) procedure were identified. The primary outcome was the 2‐year BHMR failure rate following ACLR, defined as reoperation with meniscus re‐repair or resection. Additionally, 6‐month range of motion (ROM), isokinetic knee (extension, flexion) strength, 1‐and 2‐year Knee injury and Osteoarthritis Outcome Score (KOOS), Patient‐acceptable symptom state (PASS), treatment failure (TF) were compared between the groups. Kaplan‐Meier analysis was performed to assess BHMR survival, factors associated with repair survival were analysed through Cox proportional hazard regression analysis.

**Results:**

The cohort included 159 displaced BHMRs, 120 (75.5%) underwent single‐stage surgery. The overall BHMR failure rate was 27% (43/159). The single‐stage surgery group had significantly lower failure rate (15% vs. 35.9%, *p* = 0.006). BHMT laterality, subjective (KOOS, PASS and TF) and objective (ROM, isokinetic strength) knee function did not differ significantly between the groups.

**Conclusion:**

Patients who underwent single‐stage displaced BHMR and ACLR had significantly lower BHMR failure rate compared to those who underwent two‐stage surgery. Therefore, single‐stage displaced BHMR and ACLR should be advocated, although patient‐specific factors and further prospective studies remain important considerations.

**Level of Evidence:**

Level III.

**Abbreviations**
ACL: anterior cruciate ligament
ACLR: anterior cruciate ligament reconstruction
ADL: activities of daily living
BHMR: bucket‐handle meniscus repair
BHMT: bucket‐handle meniscus tear
CI: confidence interval
HR: hazard ratio
IQR: interquartile range
KOOS: Knee Injury and Osteoarthritis Outcome Score
KOOS4: the average score of four of the five KOOS subscales, excluding ADL
LSI: limb symmetry index
PASS: patient‐acceptable symptom state
PROM: patient‐reported outcome
QOL: quality of life
ROM: range of motion
SD: standard deviation
SNKLR: Swedish National Knee Ligament Registry
Sport/Rec: sport and recreation
TF: treatment failure

## INTRODUCTION

Previous studies have reported that 44%–63% of all anterior cruciate ligament (ACL) injuries have a concomitant meniscus tear [8, 29, 33, 35]. Sustaining injuries to both the ACL and meniscus poses substantial clinical challenges, as both structures play a crucial role in maintaining knee stability and function. Combined injuries exacerbate joint instability, and in long‐term the dual injury significantly increases the risk of osteoarthritis [41], underscoring the importance of comprehensive treatment to optimise outcomes and minimise degeneration over time. Several studies have reported that meniscus repair results in less osteoarthritis [30, 43], a higher activity level and higher patient satisfaction compared to meniscus resection in the long term. Although there are certain cases where meniscus repair is not the preferred treatment, such as in highly degenerated tears, flap tears, non‐reducible bucket handle tears, or small lateral meniscus tears that do not require surgery at all [30], meniscus repair is generally recommended when feasible especially for young, active individuals.

Bucket‐handle meniscus tears (BHMTs) are a subgroup accounting for 10%–30% of all meniscus injuries [29, 58]. BHMTs are defined as longitudinal vertical tears with the inner meniscus fragment displaced into the intercondylar notch [57]. Due to the displacement of the meniscus, BHMTs often result in mechanical locking of the knee joint, instability, and pain. Thus, BHMTs should be repaired promptly whenever feasible to restore knee function.

Despite technical improvements, the failure rate of meniscal repairs remains problematic [43, 45, 48]. Displaced BHMT‐repairs (BHMRs) are associated with a failure rate ranging from 19.2% to 42.0% [23, 45, 50, 58] which is higher compared to other types of meniscus injuries. For instance, previous studies have reported failure rates of 10.6%–22.5% for longitudinal tears [20, 48, 58], 10%–13% for radial tears [5, 55] and 6.7%–20% for tears of the posterior horn [16, 23, 32]. The higher failure rate observed in BHMRs may be attributed to the greater technical challenges associated with these injuries. Bucket‐handle meniscus tears are often more difficult to reduce and suture compared to other types of meniscal injuries, potentially contributing to the increased failure rate [23]. This emphasises the need for further improvements in addressing displaced BHMTs. Over the past decade, there has been an increasing rate of meniscus repair performed in conjunction with ACLR. According to the Swedish National Knee Ligament Registry (SNKLR) [59], the frequency of meniscus repair performed in conjunction with ACLR has increased from 4% to 23% from 2005 to 2022 [59].

Patients presenting with a displaced BHMT and ACL‐injury may follow one of two possible surgical pathways as these injuries can be addressed in a single‐stage (concomitant meniscus repair and ACL reconstruction (ACLR)) or two‐stage surgery (meniscus repair followed by subsequent ACLR). Factors such as clinic's routines, surgeon's preferences, and practical considerations contribute to the course of treatment.

Most surgeons would advocate performing a single‐stage surgery repairing the meniscus and the ACL concomitantly, since it is well known that an ACL‐deficient knee is associated with a higher failure rate of meniscus sutures [4]. Some studies have suggested that early ligament surgery may lead to increased joint stiffness, advocating for delayed surgery [51], however, more recent studies suggest that this is not the case [17, 22]. To date, there is a lack of studies comparing the failure rate between single‐ and two‐stage displaced BHMR and ACLR. Furthermore, there is a lack of literature on how other important outcome measures following ACLR and displaced BHMR, such as the Knee Injury and Osteoarthritis Outcome Score (KOOS), patient‐acceptable symptom state (PASS), treatment failure (TF), knee range of motion (ROM), and isokinetic extension and flexion strength measurements differ between single‐stage and two‐stage surgery. Previous studies have either evaluated isolated ACLR or meniscus repair or compared isolated ACLR or meniscus repair to combined ACLR and meniscus repair regarding the above‐mentioned outcomes. Several of these studies have reported no significant differences in KOOS, PASS, TF, ROM and isokinetic extension and flexion between groups [6, 11, 13, 21, 31, 37, 44]. Ensuring the best surgical strategy for ACL‐injuries with associated displaced BHMTs is crucial for improving both subjective and objective patient outcomes as well as reducing meniscus repair failure rates in this patient group.

The primary aim of the current study was to compare meniscal repair failure rates between patients who underwent single‐ (concomitant BHMR and ACLR) or two‐stage (BHMR and subsequent ACLR) surgery. Another aim was to compare subjective (KOOS, PASS and TF) and objective (ROM and isokinetic strength) knee function between the single‐and two‐stage groups and assess factors associated with the survival of BHMR. It was hypothesised that single‐stage surgery would result in a lower meniscus repair failure rate compared to the two‐stage approach. It was also hypothesised that KOOS, PASS, ROM and isokinetic knee extension and flexion strength would not differ significantly between the single‐ and two‐stage groups.

## METHODS

### Patients

Ethical approval for the current retrospective study was obtained from the regional ethics committee (Karolinska Institutet, diarienummer 2016/1613‐31/32). Patient data was extracted from our clinic database. All patients who underwent single‐stage (displaced BHMR and concomitant ACLR) or two‐stage surgery (first displaced BHMR and later ACLR) at our institution between February 2015 and December 2021, with a minimum of 2‐years follow‐up after ACLR, were assessed for eligibility. Inclusion criteria were as follows: (1) BHMR and ACLR, either performed as a single‐ or two‐stage procedure, (2) BHMT intraoperatively displaced into the intercondylar notch, (3) no concomitant ligament injuries, (4) no previous meniscus or ACL surgery and (5) minimum 2‐year follow‐up after ACLR or endpoint meniscus repair failure. Missing data were reported in the respective tables for each variable. Patients were excluded only from analyses where data were missing and included in all other analyses.

### Data collection

All patients with both a meniscus injury and an ACL‐injury treated at our institution between February 2015 and December 2021 were identified. Subsequently, patients with a BHMT were selected, and their surgical records were reviewed to identify those with a displaced BHMT. Preoperative variables were collected from our clinic database and included gender, age at surgery, laterality of the injured meniscus, associated and prior knee injuries (including meniscus injuries, ACL‐injuries and other ligamentous injuries in the knee), and prior knee surgery (including meniscus surgery and ACLR). Intraoperative variables were collected from the surgical records and included assessment of the BHMT as either displaced or non‐displaced, and the type of graft used. Moreover, data on ROM and isokinetic knee extension and flexion strength measurements (limb symmetry index [LSI]) were collected from our clinic database. The KOOS was collected from the SNKLR. The database review was not blinded. Meniscus repair failure was defined as a reoperation with re‐repair or resection within the 2‐year follow‐up period, also including reoperation with re‐repair or resection during ACLR. Failures were identified through our clinic database.

### Surgical technique and rehabilitation

The surgical procedures were performed by 17 fellow‐ship trained surgeons. Standard arthroscopic anteromedial and anterolateral portals were utilised. All displaced BHMTs were prepared by rasping the tear site and adjacent synovium, followed by anatomic reduction. An all‐inside technique with FastFix (Smith & Nephew) or Fiberstitch meniscus repair devices (Arthrex) was used. The number of sutures used was determined by the requirement to adequately stabilise the repair, with a minimum of three sutures always applied. Additional inside‐out or outside‐in sutures were, according to the surgeons preference, utilised if needed in the middle third of the meniscus or if the tear extended into the anterior horn. These repairs were performed using a standard medial or lateral incision, and the tear was repaired with PDS 0 (Ethicon). Sutures were placed vertical, horizontal or oblique in the superior and inferior surfaces depending on the surgeons preference. ACLRs were performed using a single‐bundle technique. The graft was routinely fixed using a suspensory device on the femoral side and an AO bi‐cortical screw with a washer as a post on the tibial side. After meniscal repair, patients wore a postoperative hinged knee brace for 6 weeks. Flexion was increased by 30° every 2 weeks (0°–30° first and second, 0°–60° third and fourth, 0°–90° fifth and sixth week). Patients were advised to avoid weight‐bearing deep knee flexion for 4 months after meniscus repair, while weight‐bearing was otherwise allowed as tolerated. When performing a second surgery for ACLR, no brace was used unless the meniscus was re‐repaired. All patients were instructed to participate in a standardised post‐surgical rehabilitation plan. The patients were allowed to return to sports 6 months after ACLR at the earliest if the isokinetic knee muscle strength and single‐leg‐hop test criteria (LSI ≥ 90%) were met.

### ROM and isokinetic strength measurements

ROM and isokinetic knee extension and flexion strength were collected at 6 months postoperatively. ROM was measured passively using a goniometer and a deficit in flexion or extension was defined as a loss of ≥ 5°in flexion or ≥ 2° in extension compared to the uninjured leg, following the International Knee Documentation Committee guidelines [26]. Isokinetic knee extension and flexion strength was measured bilaterally at 90°/s using the Biodex System 3 (Biodex Medical Systems) [53] and reported as LSIs, a metric that has demonstrated high test‐retest reliability in previous studies [52]. The LSI is the percentage difference in strength between the injured and uninjured limb, using the uninjured limb as a reference. The test was performed in a seated position and in a ROM between 90° and 10° of knee flexion, starting with the uninjured knee first. Before the test, patients warmed up for 10 min on a stationary cycling ergometer at low resistance. A verbal description of the test was provided, and the patients completed two to three practice trials before the test. The patients then performed five maximum extension and flexion contractions with each leg, and the highest achieved values were registered [10].

### KOOS, PASS and TF

The KOOS was collected at one and two years following ACLR. The KOOS comprises 42 items divided into five subscales: Pain, Symptoms, Activities of Daily Living (ADL), Sport and Recreation (Sport/Rec) and Quality of Life (QOL). Each item is answered on a 5‐point Likert scale, then the items of each subscale are transformed to a subscale score ranging from 0 (worst) to 100 (best) [49].

To aid the interpretation of the KOOS subscale scores, the PASS and TF are commonly used. The PASS tries to identify patients who have a satisfactory subjective knee function in the operated knee, while TF can be defined as a non‐satisfactory result. PASS is assessed with a single (yes/no) question: “Taking into account all the activity you have during your daily life, your level of pain, and also your activity limitations and participation restrictions, do you consider the current state of your knee satisfactory?” [38]. In the present study, the PASS was considered to be achieved by calculating the average score of four of the five KOOS subscales, excluding ADL (KOOS4). KOOS4 was utilised as an established threshold for PASS is available for this version of the KOOS [24, 25]. PASS and TF were determined using the threshold values established by Muller et al. [38] and Granan et al. [19], respectively. The threshold value for the PASS was defined as 79 for KOOS4, whereas TF was defined as a KOOS QoL value < 44 [19].

### Statistical analysis

Normal distribution of the data was assessed using the Shapiro–Wilk test. Group comparisons between patients who underwent single‐stage (concomitant displaced BHMR and ACLR) and two‐stage surgery (displaced BHMR followed by subsequent ACLR) were made using Chi‐square test for categorical variables, Student's *t*‐test for continuous variables, and Fisher's exact test for counts expected to be smaller than five. The meniscus repair survival rate over time was analysed using Kaplan–Meier analyses. Cox proportional hazard regression analysis was used to assess factors associated with displaced BHMR failure and included treatment type (single‐stage or two‐stage), gender, age, graft type and laterality of the displaced BHMR. The proportional hazards assumption for all variables in the Cox regression model, including the multivariate analysis, was assessed using the Schoenfeld residuals test. The results indicated that the assumption was not violated for any of the covariates or the multivariate analysis. The minimum level of significance was set to *p* = 0.05. All statistical analyses were performed using RStudio version 4.3.1.

## RESULTS

### Patients

**Figure 1:**
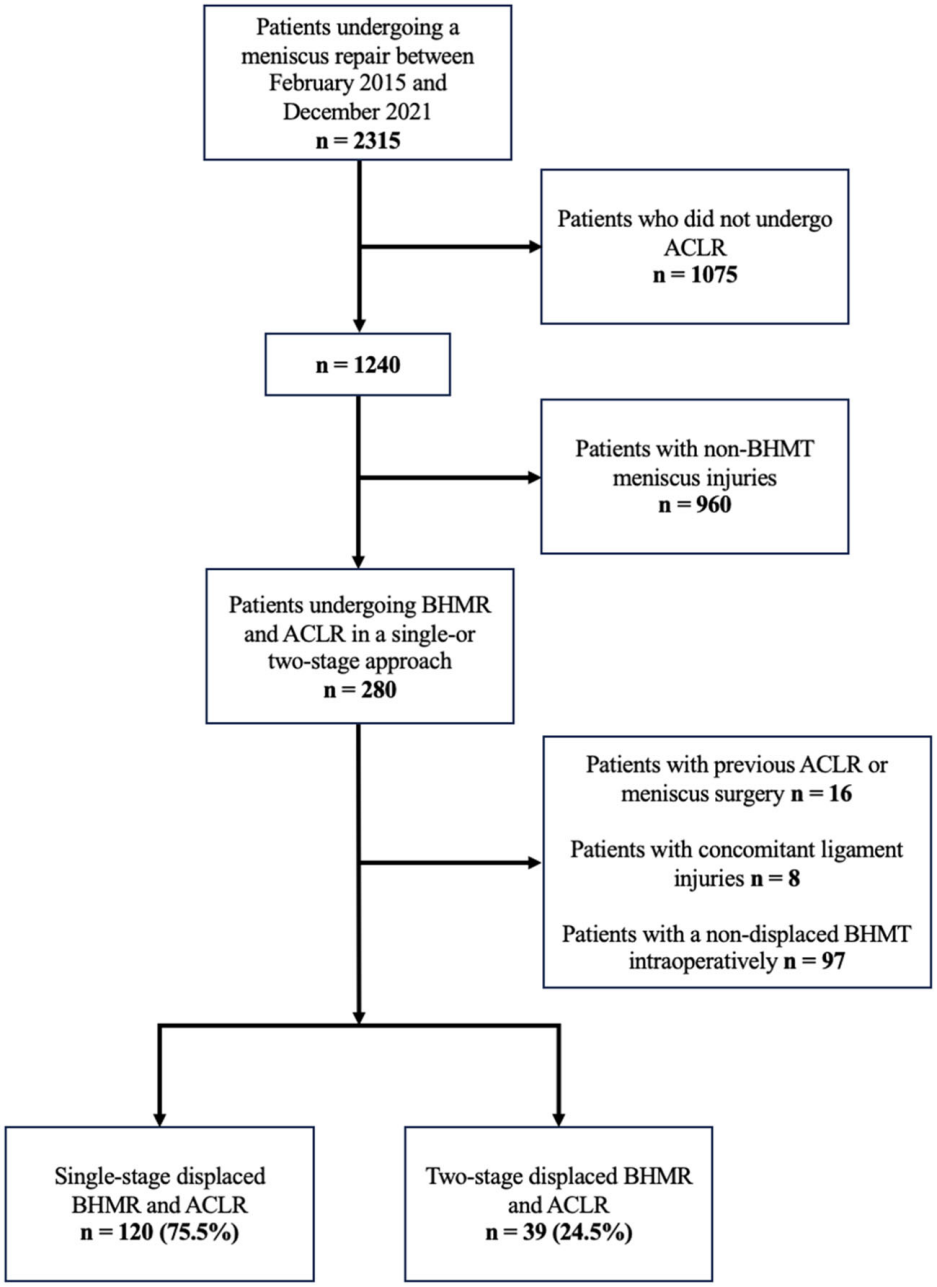
Patient flowchart. ACLR, anterior cruciate ligament reconstruction; BHMR, bucket handle meniscus repair; BHMT, bucket handle meniscus tear.

In total, 280 patients were eligible for inclusion. Of these, 121 were excluded according to the exclusion criteria. A total of 159 cases were included for analysis with 120 patients (75.5%) who underwent single‐stage surgery. One patient was included twice due to a displaced BHMR and ACLR in both knees at different time points. Patient flowchart is illustrated in Figure 1.

The mean age at surgery across the cohort was 26.8 ± 11.2 years and 76 patients (47.8%) were male. The mean follow‐up time was four years. Patient demographics and intraoperative characteristics of the study population are presented in Table 1.

**Table 1:** Patient demographics, intraoperative characteristics and BHMR‐failures.

	Treatment type		
	Single‐stage (*n* = 120)	Two‐stage (*n* = 39)	*p* value
Age at surgery (mean ± SD)	25.6 ± 11.2	30.6 ± 10.4	0.012
Gender, *n* (%)			n.s.
Male	53 (44.2)	23 (59.0)	
Female	67 (55.8)	16 (41.0)	
ACL‐injury prior to meniscus injury, *n* (%)	37 (30.8)	15 (38.5)	n.s.
Graft type *n* (%)			n.s.
Hamstrings tendon	98 (81.7)	33 (84.6)	
Quadriceps tendon	19 (15.8)	5 (12.8)	
Patellar tendon	3 (2.5)	1 (7.7)	
Meniscus laterality *n* (%)			n.s.
Medial	75 (62.5)	25 (64.1)	
Lateral	27 (22.5)	9 (23.1)	
Both	18 (15.0)	5 (12.8)	
Failure of BHMR, *n* (%)	18 (15.0)	14 (35.9)	0.006
Days to failure of BHMR (median [IQR])	336 [234–480]	89 [74–123]	<0.001
Days from ACL‐injury to meniscus repair (median [IQR])	43 [22–121]	20 [12–31]	<0.001
Days from ACL‐injury to ACLR (median [IQR])	43 [22–121]	117 [91–214]	0.021
Days from meniscus repair to ACLR (median [IQR])	0 [0–0]	89 [69–143]	‐

Abbreviations: ACL, anterior cruciate ligament; BHMR, bucket handle meniscus repair; IQR, interquartile range; n.s., not significant; SD, standard deviation.

### ROM and isokinetic strength measurements

There were no significant differences in ROM and isokinetic knee strength (extension and flexion) measurements between the study groups 6 months after ACLR (Table 2).

**Table 2:** ROM and isokinetic strength measurements 6 months after ACLR.

	Treatment group		
	Single‐stage (*n* = 120)	Two‐stage (*n* = 39)	*p* value
Flexion deficit ≥5°, *n* (%)	33 (27.5)	6 (15.4)	n.s.
Extension deficit ≥2°, *n* (%)	1 (0.8)	1 (2.6)	n.s.
Missing, *n* (%)	17 (14.2)	10 (25.6)	
Isokinetic strength, LSI ≥ 90%, *n* (%)			
Extension	19 (15.8)	5 (12.8)	n.s.
Flexion	43 (35.8)	13 (33.3)	n.s.
Missing, *n* (%)	33 (27.5)	15 (38.5)	

Abbreviations: ACLR, anterior cruciate ligament reconstruction; LSI, limb symmetry index; ROM, range of motion; SD, standard deviation; n.s., not significant.

### KOOS, PASS and TF

No significant differences were found between the study groups (single‐ or two‐stage surgery) at the 1‐ or 2‐year follow‐up for any of the KOOS subscales. Similarly, PASS and TF rates were not significantly different between the treatment groups 2 years after ACLR (Table 3).

**Table 3:** KOOS 1 and 2 years following ACLR and PASS and TF 2 years following ACLR.

	Treatment group		
	Single‐stage (*n* = 120)	Two‐stage (*n* = 39)	*p* value
1 year following ACLR (mean ± SD)			
Symptoms	93.1 ± 16.3	90.8 ± 15.6	n.s.
Pain	94.7 ± 14.7	94.3 ± 10.8	n.s.
ADL	97.9 ± 12.1	97.4 ± 8.8	n.s.
Sport/Rec	90.3 ± 21.6	83.8 ± 26.3	n.s.
QoL	87.2 ± 24.2	83.1 ± 24.4	n.s.
Missing, *n* (%)	50 (41.7)	11 (28.2)	
2 years following ACLR (mean ± SD)			
Symptoms	77.4 ± 19.5	81.1 ± 20.6	n.s.
Pain	84.4 ± 16.1	89.2 ± 13.9	n.s.
ADL	93.5 ± 11.2	95.5 ± 11.6	n.s.
Sport/Rec	70.0 ± 27.5	75.6 ± 22.4	n.s.
QoL	65.0 ± 26.4	74.3 ± 24.1	n.s.
TF, *n* (%)	11 (27.5)	2 (11.1)	n.s.
PASS (KOOS4), *n* (%)	24 (60.0)	13 (72.2)	n.s.
Missing, *n* (%)	80 (66.7)	21 (53.8)	

Abbreviations: ACLR, anterior cruciate ligament reconstruction; ADL, activities of daily living; KOOS, Knee injury and Osteoarthritis Outcome Score; KOOS4, the average patient score calculated using the KOOS subscales Symptoms, Pain, Sport/Rec and QoL; n.s., not significant; PASS, patient‐acceptable symptom state; QoL, knee‐related quality of life; SD, standard deviation; Sport/Rec, sport and recreation; TF, treatment failure.

### Meniscus repair failure

**Figure 2:**
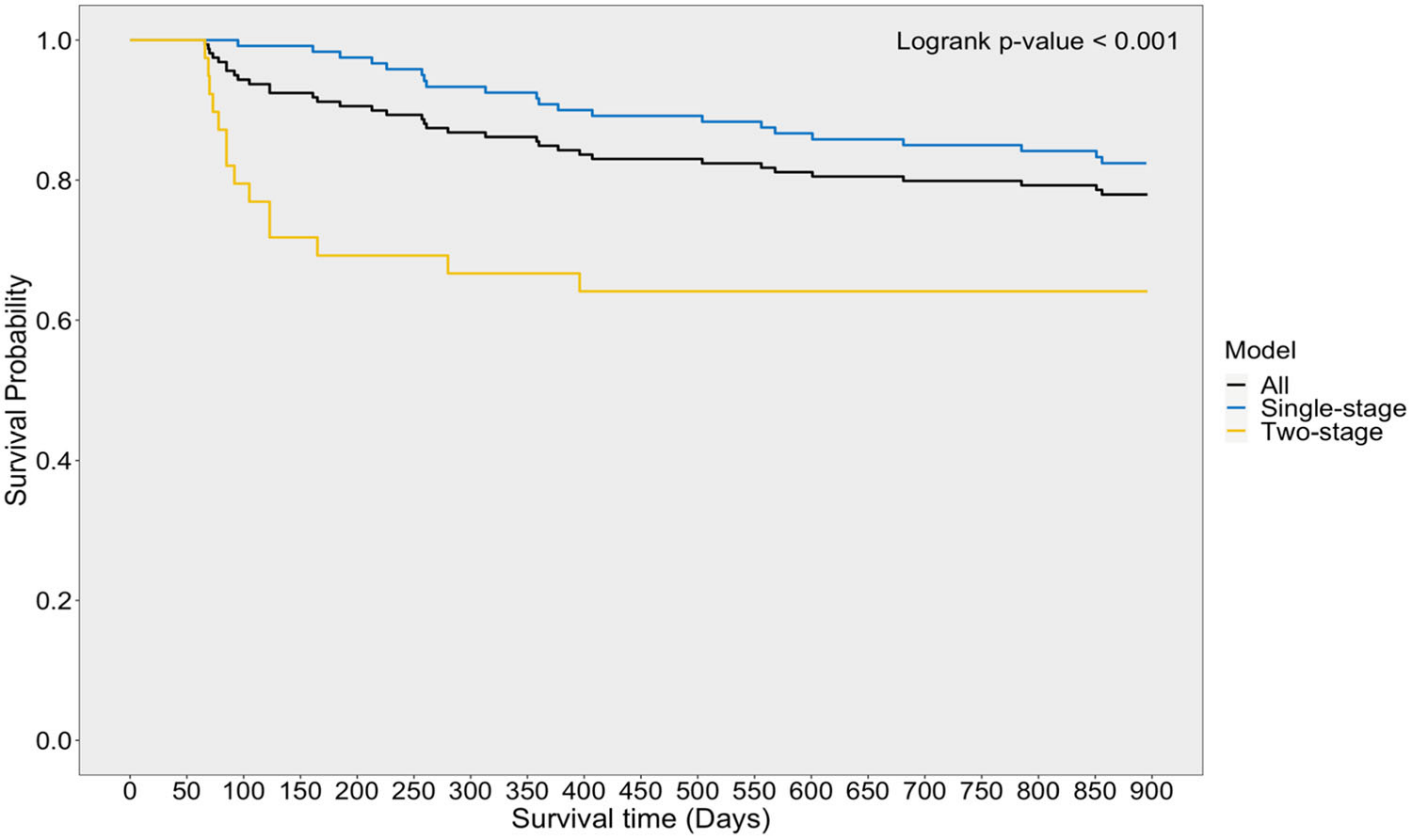
Survival function for single‐and two‐stage displaced BHMR and ACLR. ACLR, anterior cruciate ligament reconstruction; BHMR, bucket handle meniscus repair.

During follow‐up 32 displaced BHMRs (20.1%) failed. The single‐stage surgery group had 15.0% (18/120) failures with a median time to failure of 336 [234–480] days, which was significantly lower than that of the two‐stage surgery group which had 35.9% (14/39) failures with a median time to failure of 89 [74–123] days. The 14 displaced BHMR failures in the two‐stage group had their meniscus reoperation performed during the ACLR. Survival curves comparing meniscus repair survival in the single‐and two‐stage surgery groups are presented in Figure 2, and Cox proportional hazard regression analysis is presented in Table 4.

**Table 4:** Factors associated with BHMR failure in Cox proportional hazard regression analysis.

	BHMR failure (*n* = 32)					
	Univariate			Multivariate		
Variable	HR	95% CI	*p* value	HR	95% CI	*p* value
Treatment type						
One session (ref)		1			1	
Two sessions	1.56	2.13–10.71	<0.001	10.60	2.54–44.27	0.001
Gender						
Female (ref)		1			1	
Male	0.79	0.37–1.68	n.s.	0.64	0.26–1.56	n.s.
Age						
<20 (ref)		1			1	
20–30	1.57	0.73–3.35	n.s.	0.92	0.21–4.10	n.s.
31–40	0.77	0.36–1.65	n.s.	1.96	0.31–12.50	n.s.
>40	2.12	0.71–6.33	n.s.	1.09	0.24–4.87	n.s.
Graft type *n* (%)						
Hamstrings tendon		1			1	
Quadriceps tendon	0.93	0.40–2.21	n.s.	0.57	0.55–5.68	n.s.
Patellar tendon	3.40	1.87–483.27	0.02	2.86	0.57–542.01	n.s.
Meniscus suture						
Lateral (ref)		1			1	
Medial	2.60	0.13–1.11	n.s.	2.06	0.14–1.73	n.s.
Both	1.34	0.54–3.34	n.s.	2.20	0.80–6.07	n.s.

Abbreviations: ACLR, anterior cruciate ligament reconstruction; BHMR, bucket handle meniscus repair; CI, confidence interval; HR, hazard ratio; n.s., not significant.

## DISCUSSION

The most important finding of the present study was that, in accordance with our hypothesis, two‐stage displaced BHMR and ACLR was associated with a significantly higher meniscus repair failure rate compared with single‐stage surgery. Laterality of the BHMT, subjective (KOOS, PASS and TF) and objective (ROM and isokinetic strength measurements) knee function measurements did not differ significantly between the single‐and two‐stage surgery groups. The single‐stage group was significantly younger than the two‐stage group, however, the Cox proportional hazard regression analysis did not find age to be associated to BHMR failure.

It should be noted that the 14 displaced BHMR failures in the two‐stage group had their meniscus reoperation performed during the ACLR procedure. It is unclear whether these patients exhibited symptoms of meniscus suture failure, as was observed with the displaced BHMR failures in the single‐stage group, or if the failures were accidentally discovered during ACLR. This distinction has important clinical implications, as asymptomatic failures may not require immediate intervention, altering how outcomes are interpreted and potentially influencing surgical decision‐making. A previous study by Asahina et al. [3], which evaluated 98 meniscal repairs via second‐look arthroscopy in patients who underwent both ACLR and meniscus suture, reported that 15% of clinically asymptomatic patients had a meniscus suture failure at an average of 16 months following ACLR. This suggests a potential overestimation of meniscus suture failures in the two‐stage group. Furthermore, the timing of the meniscus reoperation in the two‐stage group may have influenced the observed number of days to BHMR failure, as the reoperations were performed concurrently with the ACLR. To address these issues, future prospective studies should clearly specify the reason for reoperations.

The literature is inconsistent regarding the outcomes of meniscus repairs performed in isolation or in combination with ACLR. Some studies found a higher healing rate of meniscus repair performed with concomitant ACLR [4, 14, 48], whereas others found no differences in failure rates between isolated meniscus repair and meniscus repair performed in conjunction with ACLR [6, 36, 56, 58]. However, these studies compared patients with isolated meniscus tears to those with concomitant meniscus tear and ACL‐injury. The current study found that displaced BHMRs performed concomitant to an ACLR results in fewer failures, suggesting that BHMRs might heal better when performed concomitant to ACLR. Several hypotheses have been proposed to explain why meniscal healing may be more effective when repaired concurrently with ACLR. One hypothesis is that the abundance of blood and growth factors due to the drilling of bone tunnels during ACLR foster an environment that enhances meniscal healing [18]. Previous studies have explored methods to replicate this favourable biological environment in cases of isolated meniscus tears. One such study reported a significantly lower failure rate at two years post‐surgery in patients who received an intra‐repair site injection of platelet‐rich plasma [28]. Another possible explanation is that an extended rehabilitation period, combined with increased caution in physical activities during the healing phase contributes to improved outcomes in meniscus repairs [7, 48]. The menisci are important secondary stabilisers of the knee. The medial meniscus is an important restraint of anterior tibial translation [9, 12], and the lateral meniscus is an important restraint of rotational laxity [39]. In ACL‐ and meniscus‐deficient knees, if ACLR is not performed concurrently with meniscus repair, the meniscus will experience significantly increased stress, potentially leading to failure [2, 42].

Given the critical role of the meniscus in the knee, the timing of surgical interventions becomes crucial. Although there is no clear consensus on whether single‐stage or two‐stage surgery is preferable for managing BHMR and ACLR, most surgeons would opt to perform both surgeries concomitantly if feasible. However, in cases such as an acute ACL‐injury with a bucket‐handle tear, some surgeons may prefer to allow the knee to reduce swelling before proceeding with ACLR. In this study, the median time from injury to surgery was 43 days for the single‐stage group and 20 days for the first surgery in the two‐stage group (*p* < 0.001). While the single‐stage group underwent ACLR significantly earlier than the two‐stage group, the two‐stage group underwent displaced BHMR significantly earlier. This difference may be partly due to meniscus repair typically being a shorter and less complex procedure, making it easier to schedule. Furthermore, the single‐stage group was significantly younger than the two‐stage group which could imply a higher level of activity and a greater need for prompt intervention in this group. Both younger age and higher pre‐injury activity level may enhance the likelihood of successful rehabilitation as reported by Hamrin Senorski et al. [21], since these individuals may be better equipped to follow the rehabilitation protocol.

The association between age at surgery and meniscus repair failure rate is inconsistently reported in the literature. Some studies have reported that younger age is associated with a lower rate of meniscus repair failures, probably due to less tissue degeneration [2, 4, 34, 42]. Conversely, other studies found younger age to increase the risk of meniscus suture failure due to higher activity levels and a tendency for rapid return to sport [15, 54], and some studies found no association between age at surgery and meniscus repair failure [46, 47, 48]. The current study found no association between age at surgery and displaced BHMR failure, supporting the existing evidence indicating that age is not associated with an increased risk of meniscus suture failure. However, it should be noted that the dichotomisation in the Cox proportional hazard analysis resulted in small group sizes, which could reduce statistical power and lead to potential overestimation or underestimation of effect sizes. Therefore, these results should be interpreted with caution.

In contrast, the literature shows broad consensus on the association of meniscus laterality with meniscus suture failure. Existing literature have reported that the lateral meniscus has a superior healing potential and a lower failure rate compared to the medial meniscus [11, 27, 43], however a few studies have also failed to show a such an association [1, 40]. The current study did not reveal any significant association between the laterality of the meniscus suture and meniscus suture failure. Although the Cox proportional hazard regression analysis, as expected, showed a high HR for medial meniscus repairs indicating an increased risk of meniscus suture failure this finding was not statistically significant, possibly due to a relatively small number of failures in our cohort. Another notable finding in the Cox proportional hazard regression analysis was that the use of a patellar tendon graft in ACLR was associated with significantly fewer failures in the univariate analysis. However, this significance did not persist in the multivariate analysis. Additionally, the small number of patients receiving a patellar tendon graft (3 in the single‐stage group and 1 in the two‐stage group) and the wide confidence intervals (1.87–483.27 in the univariate analysis and 0.57–542.01 in the multivariate analysis) warrant caution in interpreting these findings.

Regarding subjective measures, no previous studies have directly compared KOOS, PASS, and TF between single‐stage and two‐stage surgeries for displaced BHMR and ACLR. In this study no significant differences were found between the single‐stage and two‐stage groups in terms of subjective measures, aligning with our hypothesis that there would be no differences between the groups. However, although not statistically significant, the two‐stage group had higher mean scores across all KOOS subscales at 2 years following ACLR compared to the single‐stage group. Additionally, the percentage of TF was more than twice as high in the single‐stage group compared to the two‐stage group (27.5% vs. 11.1%). This finding appears contradictory, as the two‐stage group also exhibited a significantly higher displaced BHMR failure rate compared to the single‐stage group. This discrepancy may be explained by the two‐stage group being significantly older and potentially less physically active than the single‐stage group, which could result in lower expectations and reduced demands on knee function. Another possible explanation is that the extent of symptoms experienced by the two‐stage group due to their failed BHMR is unknown, as the reoperation was performed during the ACLR.

Similarly, no significant differences were found in the objective measures between the single‐stage and two‐stage surgery groups. However, at 6 months following ACLR, only 15.8% and 12.8% of patients in the single‐stage and two‐stage groups, respectively, achieved an LSI > 90% for extension, while 35.8% and 33.3% of patients reached this threshold for flexion. 27.5% and 15.4% of patients in the single‐stage and two‐stage groups, respectively, had a flexion deficit, while only one patient in each group (0.8% and 2.6%, respectively) had an extension deficit. The fact that relatively few patients achieved an LSI > 90% at 6 months after ACLR aligns with previous research [10]. Cristiani et al. reported that patients undergoing ACLR with concomitant meniscus repair had reduced odds of achieving symmetrical isokinetic muscle strength at 6 months after ACLR compared to those undergoing isolated ACLR [10], indicating that a concomitant meniscus surgery presents additional challenges for these patients. Both the low number of patients achieving an LSI > 90% as well as the deficits in ROM may be attributed to the BHMR rehabilitation protocol, requiring patients to wear a hinged brace for 6 weeks and avoid weight‐bearing deep knee flexion for 4 months post‐surgery, delaying the onset of full weight‐bearing and strength training.

This study has several strengths. First, there is a lack of studies directly comparing single‐and two‐stage surgery for meniscus repair and ACLR. The present study specifically addressed this knowledge gap regarding displaced BHMR and ACLR. Second, we included a large number of displaced BHMR‐patients compared to other studies on this specific type of meniscus injury, where BHMRs are often sub‐grouped resulting in small sample sizes [6, 50, 56]. Furthermore, KOOS subscale scores were included, offering an assessment of the patients' symptoms.

## LIMITATIONS

Some limitations are present. The retrospective design presents inherent limitations including lower evidentiary quality, potential bias, misclassification, and convenience sampling. To address these challenges, future research should prioritise a prospective design. Not all patients had available KOOS, ROM or isokinetic strength measurements, which is a common issue in registry studies. The highest proportion of missing data was found for KOOS at 2 years after ACLR (53.8% for the two‐stage group and 67% for the single‐stage group), potentially affecting the interpretability of the results for this variable. However, a study by Ingelsrud et al. [24] utilising data from the Norwegian Knee Ligament Registry, showed that there was no significant difference in the postoperative KOOS between responders and non‐responders. This suggests that loss to follow‐up is not likely to have had a substantial impact on the postoperative KOOS. Furthermore, comprehensive data on the specific characteristics of the meniscus tears was not available and hence not included in this study. However, as all meniscus tears included in this study were displaced BHMTs, it is reasonable to assume they were of similar and comparable size. The surgeries were performed by 17 different fellow‐ship trained surgeons according to their preferences. However, while this may introduce some variability in the outcomes, the potential diversity of the surgical approaches reflects clinical practice and increases the external validity of the study. Age was dichotomised in the Cox proportional hazard analysis resulting in small group sizes, hence these results should be interpreted with caution. Another limitation is that patients did not undergo magnetic resonance imaging or second‐look arthroscopy to evaluate meniscus healing. In the current study, failure was defined as a reoperation with re‐repair or resection within the follow‐up period. Some might argue that this definition of failure would miss asymptomatic cases, however clinical follow‐up is the most relevant in clinical practice. It is also important to recognise, as discussed earlier, that the failures in the two‐stage group underwent their meniscus reoperation during ACLR which introduces bias and leads to a potential overestimation of meniscus suture failures in the two‐stage group. Lastly, some patients may have experienced BHMR failure and undergone reoperation at another clinic during follow‐up, which could have resulted in underestimation of the number of failed BHMRs.

## CONCLUSION

Patients who underwent single‐stage displaced BHMR and ACLR had significantly lower BHMR failure rate compared to those who underwent two‐stage surgery. Therefore, single‐stage displaced BHMR and ACLR should be advocated, although patient‐specific factors and further prospective studies remain important considerations.

## AUTHOR CONTRIBUTIONS

*Conceptualization*: All authors. *Data acquisition*: Christoffer von Essen, Carolina Kekki. *Analysis*: Carolina Kekki. *Interpretation*: All authors. *Writing–original draft preparation*: Carolina Kekki. *Writing–review and editing*: All authors. *Supervision*: Riccardo Cristiani, Anders Stålman, and Christoffer von Essen. *Final approval of manuscript*: All authors.

## CONFLICT OF INTEREST STATEMENT

The authors declare no conflicts of interest.

## ETHICS STATEMENT

Ethical approval for this study was obtained from the regional ethics committee (Karolinska Institutet, diarienummer 2016/1613‐31/32).

## Data Availability

All data and materials are available on reasonable request to the corresponding author.
