# Correction to “Single‐stage ACL reconstruction and displaced bucket handle Meniscus repair is associated with lower Meniscus repair failure rates compared to two‐stage surgery”

**DOI:** 10.1002/jeo2.70746

**Published:** 2026-08-26

Kekki C, Cristiani R, Stålman A, von Essen C. Single‐stage ACL reconstruction and displaced bucket handle Meniscus repair is associated with lower Meniscus repair failure rates compared to two‐stage surgery. *J Exp Orthop*. 2025;12(1):e70199. https://doi.org/10.1002/jeo2.70199

In Table 4, the Hazard ratio listed for the univariate analysis of treatment type is incorrectly stated as 1.56. The correct Hazard ratio should be 4.78.

We apologize for this error.

